# Objective assessment of changes in nuclear morphology and cell distribution following induction of apoptosis

**DOI:** 10.1186/1746-1596-9-92

**Published:** 2014-05-12

**Authors:** Jon R Eidet, Lara Pasovic, Rima Maria, Catherine J Jackson, Tor P Utheim

**Affiliations:** 1 Department of Medical Biochemistry, Oslo University Hospital, Oslo, Norway

## Abstract

**Background:**

To objectively measure changes in nuclear morphology and cell distribution following induction of apoptosis.

**Methods:**

A spontaneously immortalized retinal pigment epithelial cell line (ARPE-19) was cultured for three days in DMEM/F12 with 10% fetal bovine serum followed by 24 hours incubation in staurosporine to induce apoptosis. Cells that were not incubated in staurosporine served as control. Caspase-3 expression in apoptotic cells was demonstrated by quantitative immunofluorescence. Nuclei were counterstained with DAPI. Assessments of nuclear morphology and cell distribution were performed using ImageJ software. Statistical analyses included Student’s t-test and Pearson’s correlation coefficient. Nearest neighbor analysis was used to assess cell nuclei distribution.

**Results:**

Caspase-3 expression in staurosporine-incubated cells increased by 471% ± 182% compared to control (*P* = 0.014). Relative to the control, cells in the staurosporine-incubated cultures had smaller average nuclear area (68% ± 5%; *P* < 0.001) and nuclear circumference (78 ± 3%; *P* < 0.001), while nuclear form factor was larger (110% ± 1%; *P* < 0.001). Cell nuclei from the staurosporine-group (R = 1.12 ± 0.04; *P* < 0.01) and the control (R = 1.28 ± 0.03; *P* < 0.01) were evenly spaced throughout the cultures, thereby demonstrating a non-clustered and non-random cell distribution. However, the staurosporine-incubated group had a significantly lower R-value compared to the control (*P* = 0.002), which indicated a move towards cell clustering following induction of apoptosis. Caspase-3 expression of each individual cell correlated significantly with the following morphological indicators: circumference of the nucleus divided by form factor (*r* = -0.475; *P* < 0.001), nuclear area divided by form factor (*r* = -0.470; *P* < 0.001), nuclear circumference (*r* = -0.469; *P* < 0.001), nuclear area (*r* = -0.445; *P* < 0.001), nuclear form factor (*r* = 0.410; *P* < 0.001) and the nuclear area multiplied by form factor) (*r* = -0.377; *P* < 0.001).

**Conclusions:**

Caspase-3 positive apoptotic cells demonstrate morphological features that can be objectively quantified using freely available ImageJ software. A novel morphological indicator, defined as the nuclear circumference divided by form factor, demonstrated the strongest correlation with caspase-3 expression.

**Virtual Slides:**

The virtual slide(s) for this article can be found here: http://www.diagnosticpathology.diagnomx.eu/vs/3271993311662947

## Background

Based on morphology, cell death can be characterized as apoptotic or necrotic or both. Apoptotic cell death conserves the organelles and cell membrane for some time whereas the nucleus undergoes early degeneration. In necrotic cell death, however, the nucleus stays relatively intact while the cell membrane and organelles show early degeneration [1]. Typically, cap-shaped chromatin margination is one of the first signs of apoptosis [2,3]. Cytosol condensation, pyknosis and cell membrane blebbing may also be seen. The nucleus then develops several electron dense micronuclei, which are often released to the extracellular space. Finally, the cells split into numerous apoptotic bodies. In vitro, apoptotic cells undergo a late phase of necrosis characterized by early cell membrane damage [4,5]. However, pyknotic and fragmented cell nuclei are not common in necrosis.

Due to the characteristic changes in nuclear morphology during apoptosis, morphological features can be used as indicators of activation of programmed cell death. A low nuclear area factor (NAF), which was originally defined as the area of the nucleus multiplied by roundness [6], has been reported as an early sign of cell death [6-8]. Some of the latter studies employed Image Pro Plus software to allocate scores based on NAF character; a completely round cell nucleus scores 1 and less round nuclei score above 1. ImageJ software, developed by National Institutes of Health (NIH), does not calculate roundness, but instead computes form factor (in ImageJ termed ‘circularity’). Using form factor, a perfect circle scores 1 and less circular structures score between 0 and 1. Therefore, to obtain NAF with ImageJ, the nuclear area can be divided by its form factor [7]. However, some groups have calculated NAF by multiplying the nuclear area by form factor [8,9]. Nonetheless, both ways of calculating NAF have been shown to be significantly correlated with cell death. Which of these two definitions of NAF is optimal has not been reported, however. In addition, these studies did not compare the expression of apoptosis-related proteins in each cell with nuclear morphology, but rather compared average nuclei measurements in apoptosis-induced cultures with that of healthy control cultures. Comparing individual cell morphology with apoptotic phenotype could be a sensitive method to identify key morphological characteristics.

As apoptosis affects cell morphology, the spatial relationship between the cells may be altered in cultures with a higher number of apoptotic cells. Using nearest neighbor analysis, the distance from each individual cell to its nearest neighboring cell is measured to calculate an R-value [10]. An R-value of 0 denotes a completely clustered growth pattern, whereas cultures with completely random spacing between cells are given a value of 1. Given the theoretical scenario of perfectly even spacing between cells, where cells are distributed in a hexagonal pattern, the R-value becomes 2.15. However, the use of nearest neighbor analysis to investigate whether apoptotic cell cultures show specific cell distribution patterns has not been undertaken.

In the present study we investigated the association between objective changes in nuclear morphology and cell distribution following induction of caspase-3 in apoptotic cells. We found that morphological features associated with caspase-3 activation could be objectively quantified. A novel morphological indicator, defined as the circumference of the nucleus divided by form factor, demonstrated the strongest correlation with caspase-3 expression.

## Methods

### Cell culture media and reagents

Cells from the adult RPE cell line ARPE-19 were obtained from the American Type Culture Collection (ATCC) (Manassas, VA). Dulbecco’s Modified Eagle’s Medium (DMEM): Nutrient Mixture F12, fetal bovine serum (FBS), bovine serum albumin (BSA), trypsin-EDTA, phosphate-buffered saline (PBS), Triton X-100, penicillin, streptomycin, and 4′, 6-diamidino-2-phenylindole (DAPI) were provided by Sigma-Aldrich (St. Louis, MO). Nunclon Δ-surface multidishes, pipettes, and other routine plastics were supplied by VWR International (West Chester, PA). Staurosporine and the primary rabbit anti-cleaved caspase-3 (Asp 175) antibody were obtained from Cell Signaling Technology (Danvers, MA). The Cy3-conjugated goat anti-rabbit IgG secondary antibody was purchased from Abcam (Cambridge, UK).

### Cell culture

Adult human retinal pigment epithelial (ARPE-19) cells were routinely cultured in 95% air and 5% CO2 at 37°C in DMEM/F12 medium containing 10% FBS, 50 units/mL penicillin, and 50 g/mL streptomycin. The cells were seeded (5000 cells/cm2) on Nunclon Δ-surface multidishes. The culture medium was changed on the second day, and confluent cultures were obtained on the third day.

### Immunocytochemistry

Cells were cultured in 24-well multidishes as described above. Samples were subsequently prepared for immunocytochemical characterization by 15 minutes of methanol fixation at room temperature followed by 30 minutes of permeabilization and blocking in PBS containing 1% BSA and 0.2% Triton X-100. Anti-cleaved caspase-3 (1:400) antibody was diluted in blocking solution (PBS with 1% BSA). The primary antibody was omitted from the negative controls. Samples were incubated overnight at 4°C. Goat anti-rabbit Cy3-conjugated secondary antibody (diluted 1:10,000 in blocking solution) was added for one hour at room temperature. Specimens were washed for 5 minutes three times in PBS, with the addition of 1 *μ*g/mL DAPI during the last wash for cell nuclei staining. To induce caspase-3 expression cells were incubated with 1 *μ*M staurosporine for 24 hours (n = 5). Control cultures were incubated for 24 hours without staurosporine (n = 3).

The specimens were studied using a Nikon Eclipse Ti fluorescence microscope and photographed at × 200 magnification with a DS-Qi1 black-and-white camera. Photomicrographs were captured at predetermined positions in each culture using a motorized microscope stage. The exposure length and gain were maintained at a constant level for all samples, and the Cy3-fluorescence brightness of the secondary antibody was within the dynamic range of the camera.

### Analyses of cell nuclear morphology and distribution with imageJ

A macro was developed for automatic assessment of nuclear morphology with ImageJ ver. 1.45r (National Institutes of Health, Bethesda, MD). In brief, for nuclear morphology measurements, 16-bit photomicrographs of DAPI-stained nuclei were converted to 8-bit images before being auto-thresholded to binary photos using the default method of the “Make Binary” function in ImageJ. Touching cell nuclei were separated by the “Watershed” function and small fragments of nuclei were discarded on the basis of area by the “Analyze Particle” function. The latter function also provided other morphological parameters, including nuclear area, circumference and form factor.

By comparing the total number of cell nuclei obtained with and without the use of “Watershed”, an estimate of range was computed for the association percentage between cell nuclei. The lower end of the range was calculated by the following formula: (total number of cell nuclei obtained with “Watershed” – total number of cell nuclei obtained without “Watershed” + 1)/total number of cell nuclei obtained with “Watershed” ×100%; whereas the higher end of the range was computed as follows: (total number of cell nuclei obtained with “Watershed” – total number of cell nuclei obtained without “Watershed”) × 2/total number of cell nuclei obtained with “Watershed” ×100%. The rationale for using this formula was as follows: If the number of cell nuclei obtained with “Watershed” decreased by ten for example compared to without using “Watershed”, then the number of associated nuclei could be anywhere from 11 to 20 depending on whether omitting “Watershed” resulted in failure to separate 11 nuclei associated in a cluster or 20 nuclei associated as ten pairs.

In addition, we employed a “Nearest Neighbor Distance”-plugin [11] to measure the distance between each cell nucleus and its nearest nucleus (Nearest Neighbor Distance; NND). Using the binary images of DAPI-stained nuclei, this plugin calculated the distance between the centroid of each nucleus and the centroid of its nearest neighboring nucleus. The centroid of a single cell nucleus was defined as the average of the x and y coordinates of all of the pixels in the nucleus. Nearest neighbor analysis was then performed to assess patterns of cell distribution in the cultures [10]. Using this analysis, cell distribution (or cell spacing) was characterized as clustered (R = 0), random (R = 1) or even (hexagonal) (R = 2.15).

Finally, the area percentage with DAPI-stained cell nuclei (volume fraction [Vv]) in each of the binary images was calculated and compared with the mean NND of the same image.

### Caspase-3 expression quantification using imageJ

For caspase-3 quantification, an ImageJ macro was developed to measure the expression in each cell. In brief, areas around each nucleus were automatically selected in the binary DAPI-stained images. The selections were transferred to the 16-bit caspase-3-stained photomicrographs using the “Add to Manager”-function. As these selections only encircle the nuclei and not the cytosol where the caspase-3 protein is found, each selection was enlarged to enclose the cytosol by the “Enlarge”-function. Thereafter, the fluorescence intensity of caspase-3 in each cell body was measured independently. By using this method we were able to correlate the nuclear morphology measurements and the caspase-3 expression measurements within each cell.

### Statistical analysis

Student’s t-test and Pearson’s correlation coefficient were used for statistical analyses (SPSS ver. 19.0). Nearest neighbor analysis was performed to assess cell nuclei distribution. *P*-values below 0.05 were considered significant.

## Results

### Induction of caspase-3 after staurosporine-incubation

**Figure 1:**
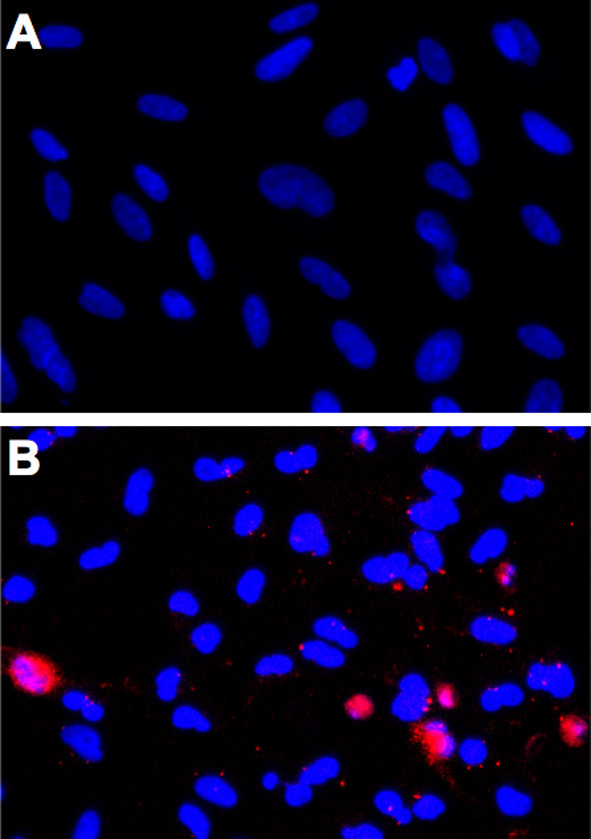
**Immunocytochemistry.** Cultured cells were incubated in 1 *μ*M staurosporine for 24 hours to induce apoptosis through caspase-3 activation (red) **(B)**. Control cells that had not been incubated in staurosporine were essentially caspase-3 negative **(A)**. Cell nuclei were counterstained with DAPI (blue). Original magnification: ×200. Photomicrographs are representative of three **(A)** and five **(B)** samples.

**Figure 2:**
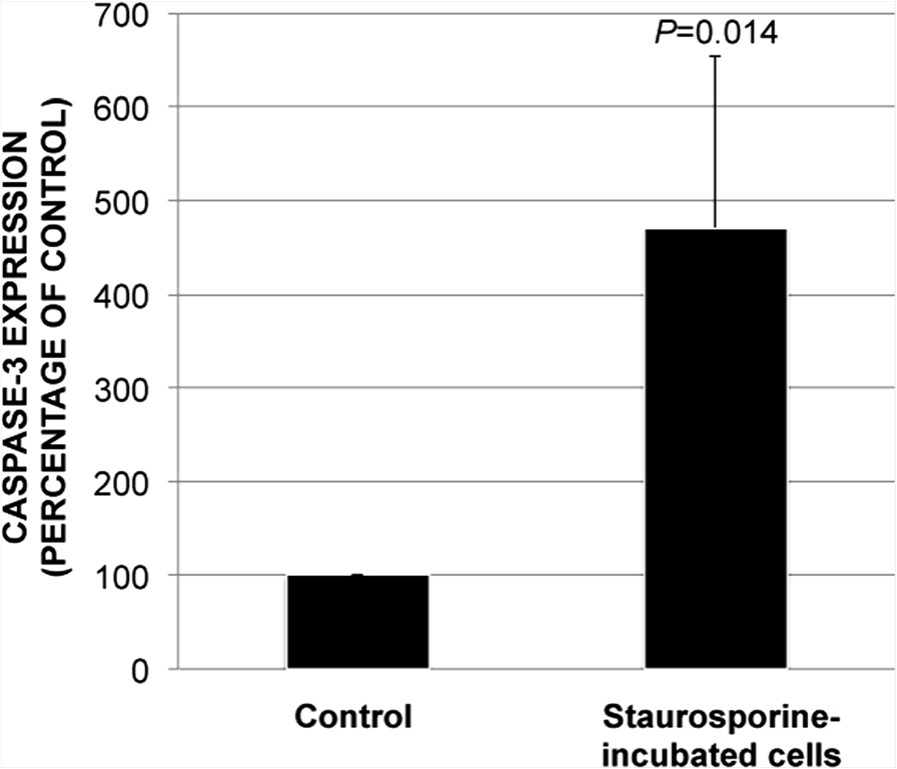
**Induction of caspase-3 after incubation in 1 *μ*M staurosporine for 24 hours.** Bar chart showing increased caspase-3 expression in staurosporine-incubated cells compared to control. Error bars represent standard deviation.

Staurosporine was used to induce apoptosis through caspase-3 expression (Figure 1B). Control cells that had not been incubated in staurosporine were essentially caspase-3 negative (Figure 1A). Caspase-3 expression in staurosporine-incubated cells increased by 471% ± 182% compared to control cells (*P* = 0.014) (Figure 2). Hence, staurosporine was effective in inducing apoptosis.

### Nuclear morphology analyses of staurosporine-incubated cells

**Figure 3:**
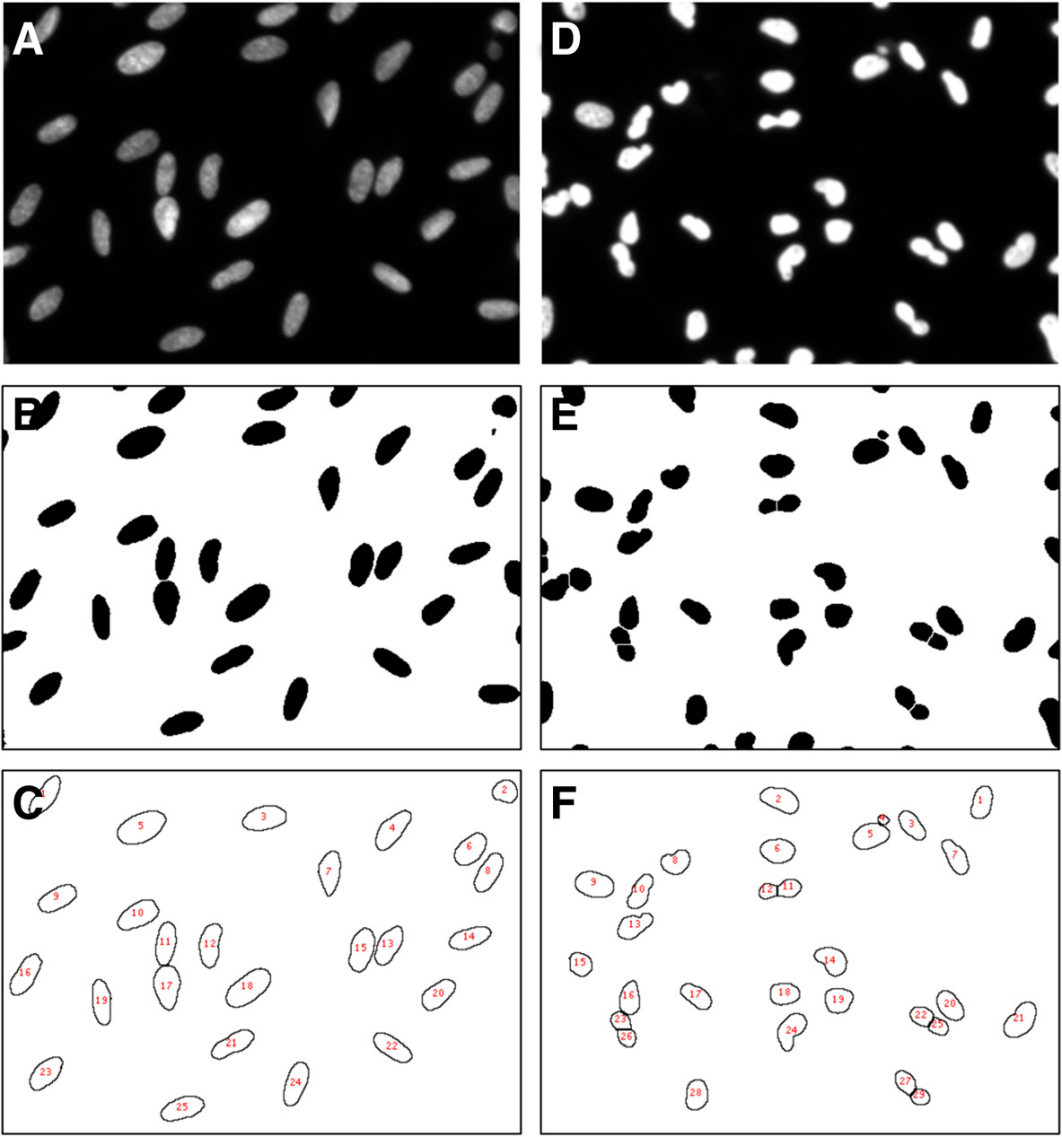
**ImageJ analyses of nuclear morphology.** To quantify the morphological changes in cell nuclei upon staurosporine incubation, cell nuclei were counterstained with DAPI and nuclear morphology was assessed with ImageJ. Photomicrographs (16-bit) of DAPI stained nuclei of control cells **(A)** were converted to 8-bit images, then auto-thresholded by the “Make Binary” function, using the default method. Touching nuclei were then separated with the “Watershed” function **(B)**. Thereafter, the “Analyze Particles” function was used to analyze nuclear morphology **(C)**. Photomicrographs of staurosporine-incubated cells were processed identically **(D-F)**. ImageJ excluded nuclei residing on the edge of the image through the “Exclude on Edges” function and small nuclear fragments were omitted from the measurements by adjusting the particle area range for the analysis. Original magnification: ×200. Photomicrographs are representative of three **(A)** and five **(B)** samples.

**Figure 4:**
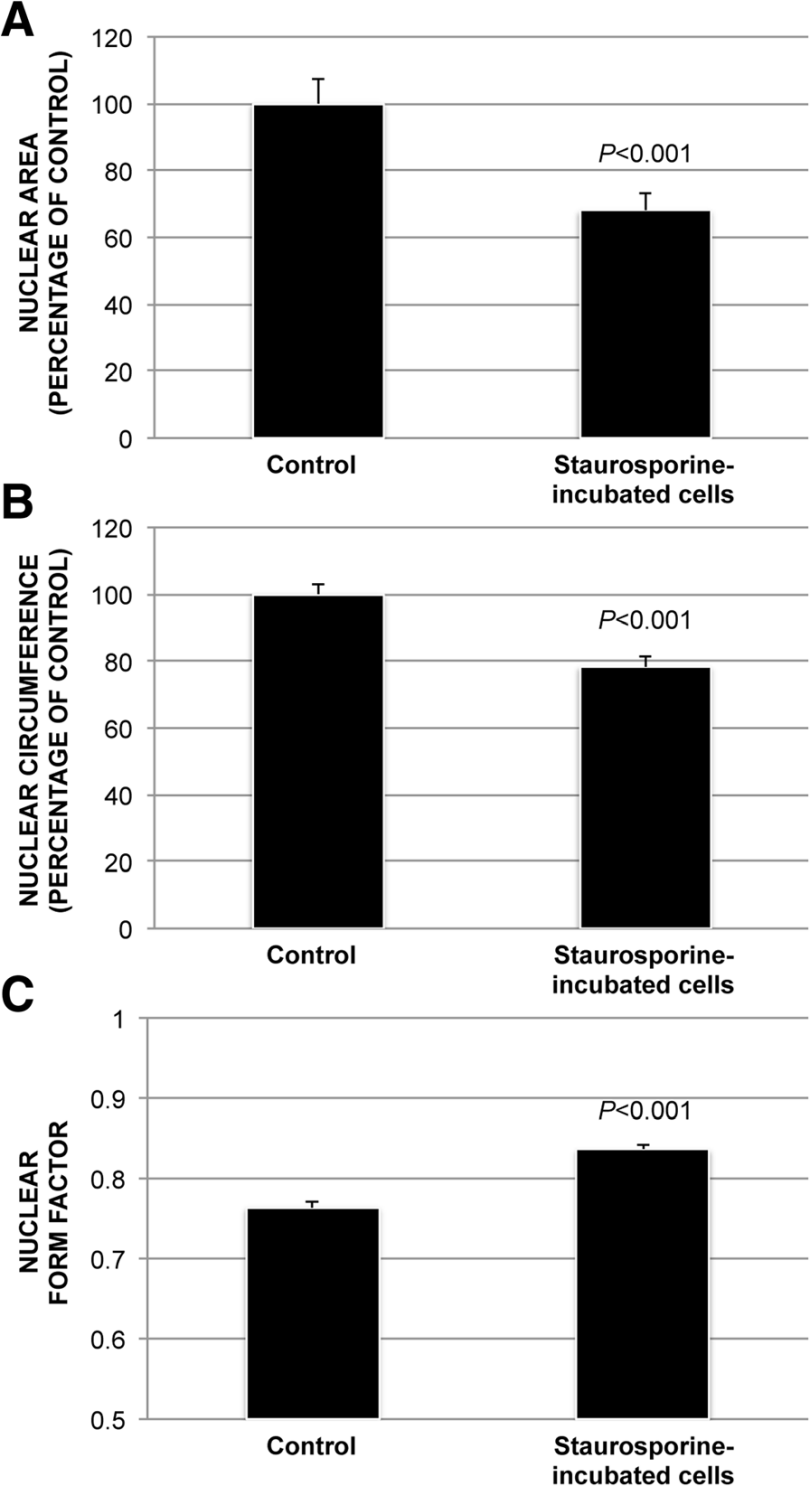
**Nuclear morphology analyses of staurosporine-incubated cells.** Bar charts demonstrate the effects of staurosporine-incubation on nuclear morphology. Relative to the control, staurosporine-incubated cultures showed a decrease in average nuclear area **(A)** and nuclear circumference **(B)**. Nuclear form factor, on the other hand, was increased in the staurosporine-exposed cultures compared to control **(C)**. Error bars represent standard deviation.

To quantify the morphological changes in cell nuclei upon staurosporine incubation, cell nuclei were counterstained with DAPI and nuclear morphology was assessed with ImageJ (Figure 3). Relative to the control, staurosporine-incubated cultures had a lower average nuclear area (68% ± 5%; *P* < 0.001) and nuclear circumference (78% ± 3%; *P* < 0.001). Compared to the control (0.76 ± 0.01), nuclear form factor increased in the staurosporine-exposed cultures (0.84 ± 0.01; *P* < 0.001) (Figure 4).

### Nearest neighbor analysis of staurosporine-incubated cells

By comparing the total number of cell nuclei obtained by ImageJ with and without the use of “Watershed”, the percentage of cell nuclei that were associated with at least one other nucleus was estimated to range between 16% and 32%.

**Figure 5:**
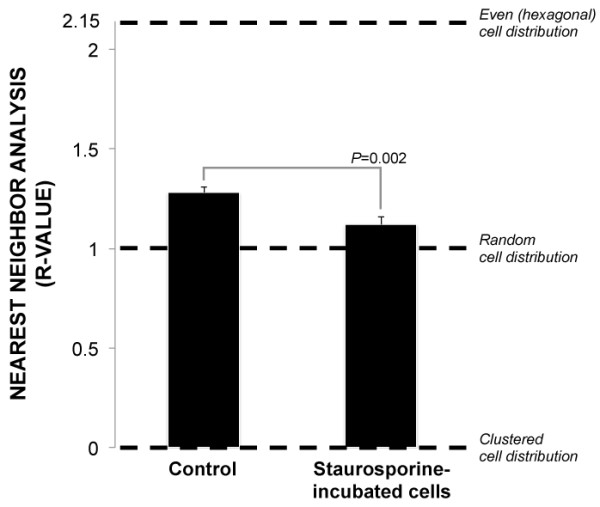
**Nearest neighbor analysis of staurosporine-incubated cells.** Nearest neighbor analysis characterizes distribution as clustered (R = 0), random (R = 1) or hexagonal (R = 2.15). This analysis was employed to assess if induction of apoptosis through staurosporine-exposure causes changes in cell distribution in culture. The R-values of the staurosporine-group were significantly higher than 1 (*P* < 0.001), indicating that cells under both conditions were not completely randomly spaced. However, the staurosporine-incubated group also had a significantly lower R-value compared to control, demonstrating that activation of apoptosis causes more uneven cell spacing. Error bars represent standard deviation.

Cell distribution in the cultures following staurosporine-incubation were investigated by employing nearest neighbor analysis [10]. With this analysis, a completely clustered cell distribution is given a value (R) of 0, a random cell distribution receives a value of 1, and a completely even (hexagonal) distribution of cells is 2.15. The R-values of the staurosporine-group (R = 1.12 ± 0.04) and the control (R = 1.28 ± 0.03) were significantly higher than 1, indicating that under both conditions cells were not completely randomly spaced (Figure 5). However, the staurosporine-incubated group had a significantly lower R-value compared to control (*P* = 0.002), demonstrating that activation of apoptosis is associated with more uneven cell spacing.

To assess whether the Vv of cell nuclei was related to the NND, the percentage of each image covered by cell nuclei was compared with the mean NND. There was no significant correlation between Vv and NND (*r* = -0.633; *P* = 0.092).

### Correlation of nuclear morphology and caspase-3 expression

**Figure 6:**
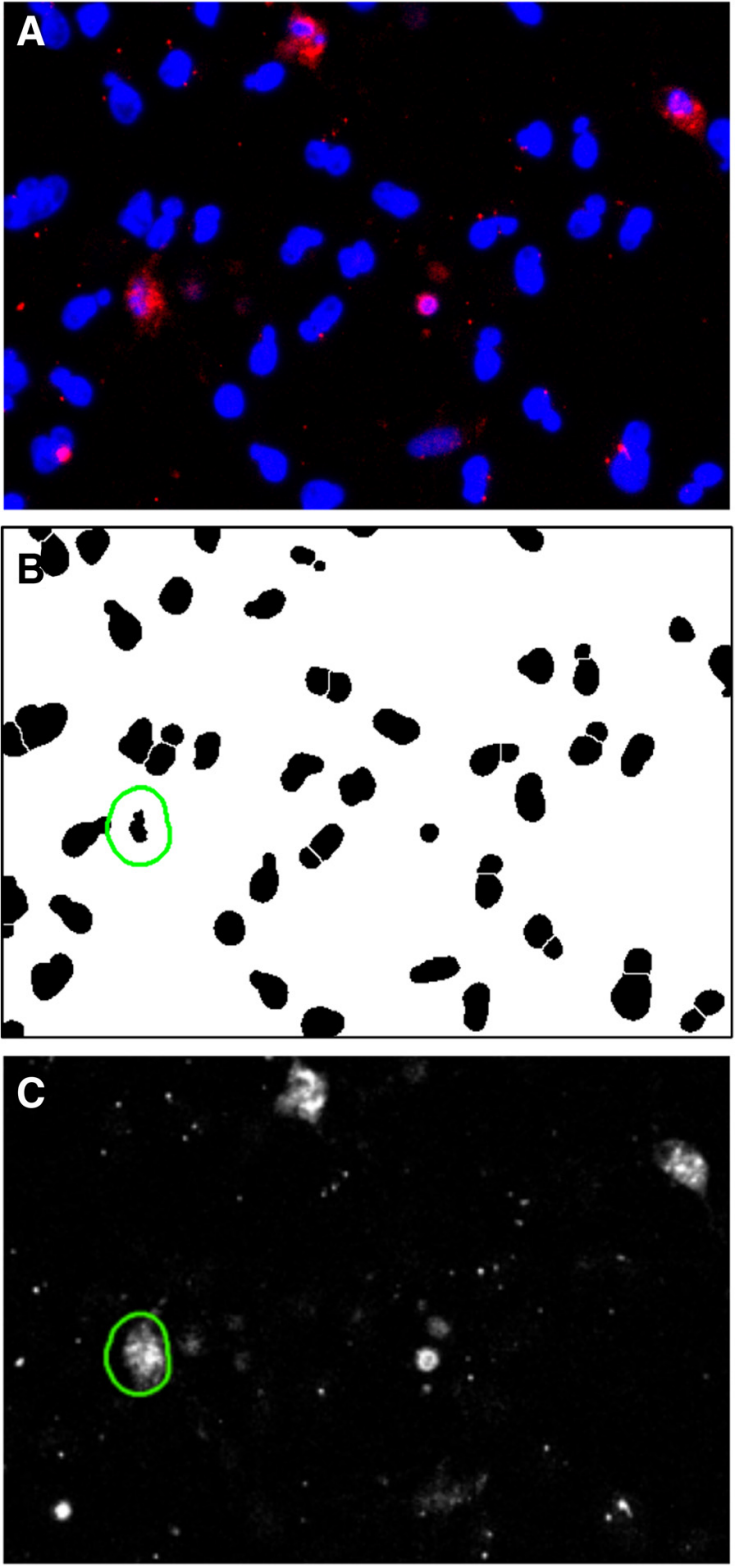
**Comparison of caspase-3 expression and nuclear morphology using ImageJ.** The caspase-3 expression of each individual cell after staurosporine-incubation was quantified and compared with the measurements of nuclear morphology to explore whether nuclear shape is correlated with apoptosis. **(A)** Photomicrograph showing staurosporine-incubated caspase-3+ (red) cells with DAPI-stained nuclei (blue). **(B)** Binary and watershed DAPI image obtained as described in Figure 3. By including the “Add to Manager” function, regions of interests (ROIs) (green) were created by ImageJ around each DAPI-stained nuclei. ImageJ enlarged the ROIs by 15 pixels to enclose most of the cell body surrounding the nucleus. **(C)** The ROIs (green), defining the area of fluorescence measurement, were converted to 16-bit gray scale caspase-3 images to measure the expression of caspase-3 in each cell after background fluorescence was removed by the ImageJ “Subtract Background” function. Caspase-3 expression in each cell was thereafter compared with nuclear morphology measurements. Original magnification: ×200.

**Figure 7:**
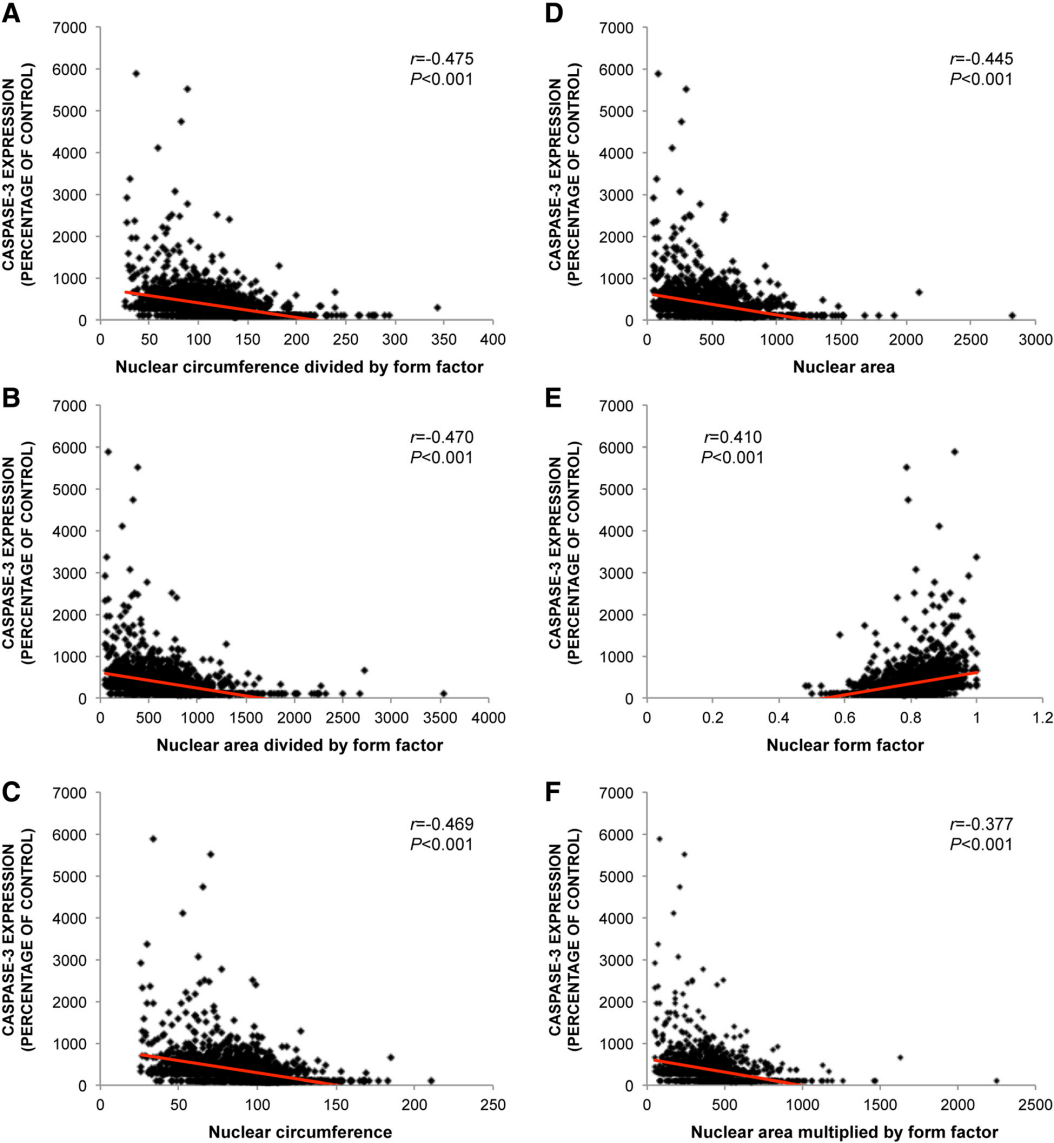
**Correlation of nuclear morphology and caspase-3 expression.** The caspase-3 expression of each individual cell was measured and compared with the measurements of nuclear morphology to explore whether nuclear shape is correlated with apoptosis. Caspase-3 expression of each individual cell correlated significantly with a morphological indicator defined as the circumference of the nucleus divided by form factor **(A)**, nuclear area factor (nuclear area divided by form factor) **(B)**, nuclear circumference **(C)**, nuclear area **(D)**, nuclear form factor **(E)** and the product of nuclear area and form factor **(F)**.

The caspase-3 expression of each individual cell was quantified and compared with the measurements of nuclear morphology to explore whether nuclear shape is correlated with apoptosis (Figure 6). Caspase-3 expression of each individual cell was found to be significantly correlated with the following morphological indicators; circumference of the nucleus divided by form factor (*r* = -0.475; *P* < 0.001), NAF (nuclear area divided by form factor) (*r* = -0.470; *P* < 0.001), nuclear circumference (*r* = -0.469; *P* < 0.001), nuclear area (*r* = -0.445; *P* < 0.001), nuclear form factor (*r* = 0.410; *P* < 0.001) and the product of nuclear area and form factor (*r* = -0.377; *P* < 0.001) (Figure 7). These results show that nuclear morphology is correlated with apoptosis. The strongest correlation was between apoptosis and the circumference of the nucleus divided by form factor.

## Discussion

In the present study we investigated the association between objective changes in nuclear morphology and cell distribution following induction of caspase-3 in apoptotic cells. We found that caspase-3-positive apoptotic cells show morphological features that can be objectively quantified. A novel morphological indicator, defined as the nuclear circumference divided by form factor, demonstrated the strongest correlation with caspase-3 expression.

Age-related macular degeneration (AMD) is a major cause of blindness in the developed world resulting from a diseased retinal pigment epithelium (RPE) [12]. For the majority of AMD patients (85%), no effective treatment alternative exists. Replacement of the RPE monolayer, however, has been proposed as a future therapy for this group [13-15]. The belief that RPE transplantation holds promise as having therapeutic benefit in the treatment of AMD builds on several studies reporting improved visual function following the restoration of the sub-retinal compartment [13]. Methods for RPE cell replacement include injection of RPE cell suspensions and transplantation of intact RPE cell sheets. Though progress has been made to improve these techniques, the production of RPE transplants is still at an experimental stage. A method for assessing quality of the transplants prior to transplantation would aid in the development of RPE replacement techniques. Quality assessment of transplants pre-operatively can be performed invasively or non-invasively. Invasive techniques may include harvesting and processing of small biopsies from the transplant for analyses of gene and protein content. Although these are powerful techniques, they risk damaging the transplant and necessitate a larger transplant due to loss of tissue during biopsy harvesting. Non-invasive techniques for quality assessment include various microscopy analyses, such as light microscopy and *in vivo* confocal microscopy (IVCM). The latter has lately been widely employed within the field of ophthalmology, aiding in the identification of putative corneal epithelial stem cell niches embedded in the ocular surface [16], keratinized surface epithelia in patients suffering from dry eye disease [17] and in the quantification of corneal endothelium density as part of quality control of corneal transplants [18]. Contrary to invasive techniques involving removal of the analyzed tissue, non-invasive image-based techniques for assessing quality of RPE cell sheets prior to transplantation would allow direct comparison of transplants pre- and post-operatively. As we did not use a non-invasive technique for obtaining images of the cell nuclei in the cultured cell sheets, the current study is a preliminary step towards the development of a method to detect apoptotic cells in RPE transplants based on cytometric measurements. Assessing cell death in RPE transplants prior to surgery could likely aid in quality selection and improve transplantation outcome.

Digital pathology has developed rapidly in recent years. Objective quantification of cell- and tissue- based measures offers the prospect of reducing bias due to subjective interpretation and help meet pathologist workload demand [19]. There are, however, several challenges with objective image quantification that need to be addressed, including image artifacts, such as blurred regions or chromatic aberrations, and batch-to-batch differences [20]. Techniques have been developed for describing images based on pixel, object, and semantic features [20]. Pixel-based image analysis derives information from features such as texture (e.g. sharpness, contrast) and color. The latter has been used to classify breast tumors through data reduction based on diffusion maps [19]. Object-level features encompass higher order features including cellular structures (e.g. nuclei, cytoplasm). Object-level information can be obtained through image segmentation. Semantic level features build on lower level features and make use of preprocessing methods, such as the bag-of-features method [21]. The latter can also include machine-learning technology. Digital pathological techniques are increasingly employed for objective assessment of whole-slide image, the latter of which is gradually becoming common clinical practice [20].

In our study, staurosporine-incubated cells differed significantly from control cells with regards to their smaller nuclear area and circumference and their higher form factor. Similar results have been reported with several other cell types [6-9,22]. The decrease in cell nuclei due to DNA loss and the increase in form factor upon initiation of apoptosis form the rationale for using NAF as a morphological indicator of apoptosis.

Both the staurosporine-exposed cultures and the control cultures exhibited a relatively even cell distribution (non-clustered and non-random) in the current study. However, the staurosporine-exposed cultures presented less even cell spacing. Our results are partly in line with a previous study in which blood-derived Jurkat cells temporarily clustered upon initiation of apoptosis [22]. However, the authors did not employ nearest neighbor analysis, but demonstrated cell clustering in photomicrographs. The lack of obvious cell clustering upon apoptosis in the current study may have been due to our use of adherent cells. One third to one sixth of the cell nuclei in our study were co-associated. These numbers are only approximate, however, as they are derived from comparing cell counts with and without the “Watershed” algorithm. Applying “Watershed” in ImageJ could, in theory, inadvertently either 1) removed some cell nuclei by separating them into small fragments that were excluded from the cell count on the basis of area; or 2) increased the number of cells by dividing large single cell nuclei into two or more fragments that were included in the cell count as separate cells. The NND, which was quantified to assess cell nuclei distribution, was defined as the distance between the centroid of each individual nucleus and its closest neighboring nucleus. From this it follows that the NND of associated nuclei would be approximately equal to the sum of the radii of the two nuclei. Thus, this may have affected the computation of cell nuclei distribution, giving a tendency for a somewhat larger NND with larger nuclei.

The use of immunocytochemistry allows for the comparison of protein expression and morphology in single cells. In addition, by employing quantitative immunofluorescence the relative protein expression can be objectively measured. In the current study, we compared single cell caspase-3 expression with the same cell’s morphology measurements. This allowed a more sensitive analysis of the relationship between cell morphology and phenotype than merely comparing the mean phenotype of entire cell cultures/samples [7-9,22]. Using this method, we found that caspase-3 expression in apoptotic cells showed the highest correlation with a morphologic indicator defined as the nuclear circumference divided by form factor. We also found that the NAF, when computed by its original formula [6]: nuclear area divided by form factor, had greater correlation with caspase-3 expression than when calculating the NAF by the formula: nuclear area multiplied by form factor. In fact, the morphological indicators nuclear circumference, area and form factor *alone* had greater correlation with caspase-3 expression than nuclear area multiplied by form factor.

We used DAPI as nuclear stain in the current study due to its simple staining protocol, high specificity and availability for quantitative analyses [23]. DAPI can be used with live and fixed cells [23], and its fluorescence increases 20-fold upon binding to DNA [24]. However, a possible disadvantage of using DAPI is the increase in glare from intensely bright DAPI-stained nuclei. Glare can affect nuclear measures and must be taken into consideration when interpreting the results in the current study. Techniques for avoiding bias due to glare have been described elsewhere [25]. Feulgen stain is considered the gold standard for DNA image cytometry [26]. It has been widely used for objective DNA quantification as the amount of stain seen in the nucleus directly correlates with DNA content [27]. However, the staining protocol is comparatively longer and is more laborious compared to using DAPI. The latter has been shown to yield reliable measurements of staining intensity and nuclear area size [28]. Moreover, DAPI gives similar results as the conventional Feulgen staining [28], making DAPI a good candidate for use in cytometric analyses.

In this study, we performed both random and stratified sampling [29,30]. The former was used when 8-bit images were thresholded to binary images, thereby segmenting the images into figure (nuclei) and background (space between nuclei) [29]. Stratified sampling was done when measuring fluorescence intensity of the Cy3-conjugated caspase-3 antibody in the vicinity of each cell nucleus. For closely associated nuclei, we cannot fully exclude the possibility of biased measurements of caspase-3 expression due to overlapping cells. However, this should not have affected the results significantly, as we mainly used monolayer cultures. We employed the default method defined in ImageJ for auto-thresholding each photomicrograph to create binary images. There are, however, at least 16 different thresholding algorithms available in ImageJ, the use of which may have given different results.

## Conclusion

We conclude that caspase-3 positive apoptotic cells demonstrate morphological features that can be objectively quantified using freely available ImageJ software. A novel morphological indicator, defined as the circumference of the nuclei divided by form factor, demonstrated the strongest correlation with caspase-3 expression. Objective comparison of individual cell phenotype and morphology using ImageJ as presented in the current study can potentially contribute to the development of defined morphological indicators of activation of certain cellular pathways.

## Competing interests

The authors declare that they have no competing interests.

## Authors’ contribution

JRE designed the study and drafted the manuscript. LP and RM performed immunofluorescence. CJJ drafted the manuscript. TPU drafted the manuscript. All authors read and approved the final manuscript.

## References

[B1] Vitale M Zamai L Mazzotti G Cataldi A Falcieri E Differential kinetics of propidium iodide uptake in apoptotic and necrotic thymocytes Histochemistry 1993 100 223 229 10.1007/BF00269095 8244773

[B2] Falcieri E Zamai L Santi S Cinti C Gobbi P Bosco D Cataldi A Betts C Vitale M The behaviour of nuclear domains in the course of apoptosis Histochemistry 1994 102 221 231 10.1007/BF00268899 7868364

[B3] Stuppia L Gobbi P Zamai L Palka G Vitale M Falcieri E Morphometric and functional study of apoptotic cell chromatin Cell Death Differ 1996 3 397 405 17180110

[B4] Buja LM Eigenbrodt ML Eigenbrodt EH Apoptosis and necrosis: basic types and mechanisms of cell death Arch Pathol Lab Med 1993 117 1208 1214 8250690

[B5] Ziegler U Groscurth P Morphological features of cell death News Physiol Sci 2004 19 124 128 15143207 10.1152/nips.01519.2004

[B6] Daniel B DeCoster MA Quantification of sPLA2-induced early and late apoptosis changes in neuronal cell cultures using combined TUNEL and DAPI staining Brain Res Brain Res Protoc 2004 13 144 150 10.1016/j.brainresprot.2004.04.001 15296851

[B7] DeCoster MA The nuclear area factor (NAF): a measure for cell apoptosis using microscopy and image analysis Modern Res and Educ Topics in Microsc 2007 1 378 384

[B8] Helmy IM Azim AM Efficacy of ImageJ in the assessment of apoptosis Diagn Pathol 2012 7 15 10.1186/1746-1596-7-15 22309648 PMC3307432

[B9] Afifi NS Abdel-Hamid ES Baghdadi HM Mohamed AF Nuclear Area Factor as a Novel Estimate for Apoptosis in Oral Squamous Cell Carcinoma -Treated Cell Line: A Comparative in-vitro Study with DNA Fragmentation Assay J Clinic Experiment Pathol 2012 2 107 doi:10.4172/2161-0681.1000107

[B10] Clark P Evans F Distance to nearest neighbor as a measure of spatial relationships in populations Ecol Soc of Am 1954 35 445 453

[B11] MediaWiki https://icme.hpc.msstate.edu/mediawiki/index.php/Nearest_Neighbor_Distances_Calculation_with_ImageJ

[B12] Jager RD Mieler WF Miller JW Age-related macular degeneration N Engl J Med 2008 358 2606 2617 10.1056/NEJMra0801537 18550876

[B13] da Cruz L Chen FK Ahmado A Greenwood J Coffey P RPE transplantation and its role in retinal disease Prog Retin Eye Res 2007 26 598 635 10.1016/j.preteyeres.2007.07.001 17920328

[B14] Sheridan CM Mason S Pattwell DM Kent D Grierson I Williams R Replacement of the RPE monolayer Eye 2009 23 1910 1915 10.1038/eye.2008.420 19169229

[B15] Lund RD Adamson P Sauvé Y Keegan DJ Girman SV Wang S Winton H Kanuga N Kwan ASL Beauchène L Zerbib A Hetherington L Couraud P-O Coffey P Greenwood J Subretinal transplantation of genetically modified human cell lines attenuates loss of visual function in dystrophic rats Proc Natl Acad Sci U S A 2001 98 9942 9947 10.1073/pnas.171266298 11504951 PMC55557

[B16] Zarei-Ghanavati S Ramirez-Miranda A Deng SX Limbal lacuna: a novel limbal structure detected by in vivo laser scanning confocal microscopy Ophthalmic Surg Lasers Imaging 2011 42 e129 131 22150603 10.3928/15428877-20111201-07 PMC8819905

[B17] Kojima T Matsumoto Y Dogru M Tsubota K The application of in vivo laser scanning confocal microscopy as a tool of conjunctival in vivo cytology in the diagnosis of dry eye ocular surface disease Mol Vis 2010 16 2457 2464 21139693 PMC2994731

[B18] Jonuscheit S Doughty MJ Ramaesh K In vivo confocal microscopy of the corneal endothelium: comparison of three morphometry methods after corneal transplantation Eye 2011 25 1130 1137 10.1038/eye.2011.121 21660067 PMC3178261

[B19] Belhomme P Oger M Michels JJ Plancoulaine B Herlin P Towards a computer aided diagnosis system dedicated to virtual microscopy based on stereology sampling and diffusion maps Diagn Pathol 2011 6 1 S3 10.1186/1746-1596-6-3 21489198 PMC3073221

[B20] Kothari S Phan JH Stokes TH Wang MD Pathology imaging informatics for quantitative analysis of whole-slide images JAMIA 2013 20 1099 1108 23959844 10.1136/amiajnl-2012-001540 PMC3822114

[B21] Cruz-Roa A Caicedo JC Gonzalez FA Visual pattern mining in histology image collections using bag of features Artif Intell Med 2011 52 91 106 10.1016/j.artmed.2011.04.010 21664806

[B22] DeCoster MA Maddi S Dutta V McNamara J Microscopy: Science, technology, applications and education. vol 2 2010 Badajoz, Spain: Formatex Research Center 836 843

[B23] Tarnowski BI Spinale FG Nicholson JH DAPI as a useful stain for nuclear quantitation Biotech Histochem 1991 66 297 302 1725854

[B24] Chazotte B Labeling nuclear DNA using DAPI Cold Spring Harb Protoc 2011 2011 pdb prot5556 21205856 10.1101/pdb.prot5556

[B25] Haroske G Meyer W Theissig F Kunze KD Increase of precision and accuracy of DNA cytometry by correcting diffraction and glare errors Anal Cell Pathol 1995 9 1 12 7577750

[B26] Biesterfeld S Beckers S Del Carmen Villa Cadenas M Schramm M Feulgen staining remains the gold standard for precise DNA image cytometry Anticancer Res 2011 31 53 58 21273580

[B27] Bacus SS Goldschmidt R Chin D Moran G Weinberg D Bacus JW Biological grading of breast cancer using antibodies to proliferating cells and other markers The Am J of Pathol 1989 135 783 792 PMC1880091 2817079

[B28] Kayser K Baumgartner K Gabius HJ Cytometry with DAPI-stained tumor imprints: a reliable tool for improved intraoperative analysis of lung neoplasms Anal Quant Cytol Histol 1996 18 115 120 8744500

[B29] Kayser K Radziszowski D Bzdyl P Sommer R Kayser G Towards an automated virtual slide screening: theoretical considerations and practical experiences of automated tissue-based virtual diagnosis to be implemented in the Internet Diagn Pathol 2006 1 10 10.1186/1746-1596-1-10 16764733 PMC1524814

[B30] Kayser K Schultz H Goldmann T Gortler J Kayser G Vollmer E Theory of sampling and its application in tissue based diagnosis Diagn Pathol 2009 4 6 10.1186/1746-1596-4-6 19220904 PMC2649041

